# Predictors of substantial improvement in physical function six months after lumbar surgery: is early post-operative walking important? A prospective cohort study

**DOI:** 10.1186/s12891-019-2806-7

**Published:** 2019-09-11

**Authors:** Sarah J. Gilmore, Andrew J. Hahne, Megan Davidson, Jodie A. McClelland

**Affiliations:** 1 St Vincent’s Private Hospital Melbourne, 59 Victoria Parade, Fitzroy, VIC 3065 Australia; 2 La Trobe University, Plenty Rd, Bundoora, Melbourne, VIC 3086 Australia

**Keywords:** Physical therapy, Physical activity, Lumbar surgery, Lumbar fusion, Discectomy, Laminectomy

## Abstract

**Background:**

Resuming walking after lumbar surgery is a common focus of early post-operative rehabilitation, however there is no knowledge about whether increased walking is associated with better functional outcomes. This study aimed to determine whether time spent walking in the week after lumbar surgery, along with co-morbidities, pre-operative pain duration, pre-operative physical activity or function, or surgical variables predict substantial improvement in physical function six months after lumbar surgery.

**Methods:**

A prospective cohort study design was utilized. Participants undergoing lumbar surgery (discectomy, decompression, fusion) were recruited between April and November 2016. Predictor variables were collected pre-operatively (age, sex, smoking status, obesity, diabetes, depression, anxiety, pre-operative pain duration, neurological deficit, physical activity levels, mobility restriction, function) and early post-operatively (post-operative walking time, surgical procedure, single/multi-level surgery). Outcome variables (physical function, back pain and leg pain severity) were measured pre-operatively and six-months post-operatively. Logistic regression analysis was used to establish prediction of substantial improvement in outcome at six months.

**Results:**

Participants (*N* = 233; 50% female; age 61 (SD = 14) years) who walked more in the first post-operative week were more likely to have substantially improved function on the Oswestry Disability Questionnaire at six months (OR 1.18, 95%CI 1.02–1.37), as were participants with < 12 months pre-operative pain (OR 2.71, 95%CI 1.28–5.74), and those with lower pre-operative function (OR 4.02, 95%CI 2.33–6.93). Age < 65 years (OR 2.36, 95%CI 1.14–4.85), and < 12 months pre-operative pain (OR 3.52 95%CI 1.69–7.33) predicted substantial improvement on the SF-36 Physical Component Summary. There were no significant predictors for substantial improvement in either leg or back pain.

**Conclusions:**

Walking time in the week after lumbar surgery is one of several predictors of substantial improvement in function at six months. Further research is required to determine whether intervention designed to increase walking early after lumbar surgery results in improved longer-term recovery of function.

**Trial registration:**

Australian New Zealand Clinical Trials Registry (ANZCTR), registration number 12616000747426. Retrospectively registered on the 7th of June 2016.

**Electronic supplementary material:**

The online version of this article (10.1186/s12891-019-2806-7) contains supplementary material, which is available to authorized users.

## Background

The number of surgical procedures performed for the management of lumbar spinal conditions has increased considerably over the previous two decades [1–3]. While surgical techniques have advanced over this time, whether surgical intervention leads to improved patient outcome remains inconclusive [4–6], with up to 40% of patients reporting no improvement in symptoms following surgery [7]. It may be possible to improve the success of surgical intervention through rehabilitation programs specifically designed to optimise post-operative recovery. However, there has been very little research investigating the effectiveness of rehabilitation after lumbar spine surgery [8, 9].

The majority of patients undergoing lumbar surgery in Australia and the UK are seen by a physiotherapist during their hospital admission. While the focus of early rehabilitation is commonly on resuming walking [10–12], walking time in the post-operative period has not previously been evaluated for its association with longer term outcomes in people undergoing lumbar surgery. A number of other factors have been shown to predict outcomes following lumbar surgery including age [13, 14], sex [13, 15], smoking status [16] obesity [13], diabetes [17], depression [18], anxiety [13], symptom duration [15], and pre-operative disability [13, 14, 16]. To further the current understanding about recovery after lumbar surgery is important that additional potential predictive factors, such as walking time in the immediate post-operative period, are evaluated relative to these existing predictors of surgical outcome.

Objective measurement using accelerometry is a valid [19] and increasingly common method of quantifying walking and physical activity after lumbar surgery. Recent research indicates that patients do very little walking in the first post-operative week [20], a trend that continues for up to two years after surgery [21]. It also appears that patients perform significantly less walking six months after lumbar surgery than they have the capacity to do [22], suggesting that it may be possible to increase walking time through targeted intervention. This discrepancy further supports for the need to investigate whether increased walking time is associated with better post-operative outcomes.

The aim of this study, as described in the published protocol [23], was to investigate which variables predict improvement of a) physical function and b) back and leg pain severity six months after lumbar spinal surgery, with a focus on walking time early after surgery.

## Methods

### Study design

A prospective cohort study design was used. Ethics approval was obtained from the St Vincent’s Hospital Melbourne Human Research Ethics Committee (Reference: LRR 098/15).

### Participants

Participants were recruited from St Vincent’s Private Hospital Melbourne (SVPHM). Patients aged 18 years or older, admitted for surgical management of a disc prolapse, degenerative disc disease, lumbar spinal stenosis and/or degenerative spondylolysthesis were invited to participate. Patients were excluded if surgery was for the management of fractures or tumours, if they were unable to provide informed consent, or if they had a co-existing neurological or musculoskeletal condition resulting in impaired physical function. There were no exclusion criteria based on the duration or nature of pre-operative symptoms.

### Procedure

Potential participants were identified from the neurosurgical theatre lists of the 11 neurosurgeons performing spinal surgery at SVPHM at the time of this study. Prior to surgery, the research physiotherapist informed eligible participants about the study aims and procedures, and provided them with an information pack and pre-operative outcome measures. Written informed consent was obtained from all individual participants included in the study.

Post-operatively, participants wore an ActivPAL3 accelerometer (PAL Technologies, Glasgow, UK) to record total walking time over the first seven post-operative days, commencing at 8 am on the morning after surgery. On discharge from hospital, participants were provided with instructions to return the monitor after the recording period was completed. Six months after surgery participants were posted follow-up outcome measures to complete, which were returned to the researchers by mail.

All participants received routine post-operative physiotherapy treatment that commenced the day after surgery. This consisted of an exercise program (trunk and lower limb strengthening and stretching exercises, individualised based on patient presentation), a bed mobility and gait assessment, and a walking program. There were no post-operative restrictions on walking, all participants were encouraged to complete regular walks over a comfortable distance, increasing the duration and frequency of walking as tolerated. All participants were advised to avoid bending, lifting, or twisting for six weeks after surgery. Five of the eleven neurosurgeons advised patients to sit for no longer then 15 min at a time for six weeks, the remaining six had no post-operative sitting restrictions.

#### Predictor variables

Total walking time was measured over the first post-operative week, using the ActivPAL3 accelerometer. The ActivPAL3 has been shown to be a valid measurement tool early after lumbar spine surgery (19). In addition to post-operative walking time, an additional 14 predictor variables were included, based on previous evidence to suggest a correlation with outcome after lumbar surgery, or a strong theoretical rational for their inclusion. These variables were age, sex, current smoking status, obesity, diabetes, depression, anxiety, pre-operative pain duration, presence of a pre-operative neurological deficit, pre-operative activity level, mobility, and function, the surgical procedure performed and the number of operated vertebral levels (Table 1).

**Table 1:** Outcome and predictor variables

	Outcome measurement tool	Logistic multivariable analysis categories				
Outcome variables (Pre-operative/6 months)						
Physical function	Oswestry Disability Questionnaire (ODQ) (0–100)	1. <SCB (18.8)		2. ≥ SCB (18.8)		
	SF-36 Physical Component Summary (SF-36 PCS) (0–100)	1. <SCB (− 6.2)		2. ≥ SCB (−6.2)		
Back Pain	Numerical pain rating scale (NRPS) (0–10)	1. <SCB (2.5)		2. ≥SCB (2.5)		
Leg Pain	Numerical pain rating scale (NRPS) (0–10)	1. <SCB (2.5)		2. ≥SCB (2.5)		
Predictor variables						
Total walking time (1st 7 post-op days)	ActivPAL3 accelerometer	Hours (continuous)				
Age	–	1. < 65 years		2. ≥65 years		
Sex	–	1. Male		2. Female		
Current smoking status	–	1. Non-smoker		2. Smoker		
Obesity	Body Mass Index (BMI)	1. BMI < 30		2. BMI ≥30		
Diabetes	–	1. Not diabetic		2. Diabetic		
Depression	Patient Health Questionnaire Depression Scale (PHQ-9)	1. No depression: PHQ-9 < 10		2. Increased likelihood of depression: PHQ-9 ≥ 10		
Anxiety	Generalised Anxiety Disorder 7 Item Scale (GAD-7)	1. No anxiety: PHQ-9 < 10		2. Increased likelihood of anxiety: PHQ-9 ≥ 10		
Pre-operative pain duration	–	1. < 12 months		2. ≥12 months		
Neurological deficit	Self-report	1. No deficit		2. Sensory/motor deficit		
Pre-operative activity	International Physical Activity Questionnaire Short Form (IPAQ-SF)	1. Moderate/high activity		2. Low activity		
Pre-operative mobility	ODQ, Section 4 (restricted mobility: score ≥ 3: unable to walk more than 500 m, or requires a stick, crutches or other support)	1. Unrestricted mobility		2. Restricted mobility		
Pre-operative function	ODQ classification (%)	1. 0–20%	2. 21–40%	3. 41–60%	4. 61–80%	5. 81–100%
Surgical procedure	–	1. Decompression		2. Discectomy		3. Fusion
Number of vertebral levels	–	1. Single level		2. Multi-level		

*SCB* Substantial Clinical Benefit

Depression was assessed using the Patient Health Questionnaire Depression Scale (PHQ-9), a valid and reliable measure of depression, that has been recommended for use in the chronic back pain population [24, 25]. Anxiety was assessed using the Generalised Anxiety Disorder 7-item Scale (GAD-7), a validated measure used to screen for, and assess the severity of anxiety [26]. Neurological deficit was assessed by participant self-report of any changes to the sensation or strength in their affected lower limb. This level of information was deemed appropriate, as any sensory or motor deficit that the participant was not aware of was unlikely to impact physical function. Pre-operative activity was assessed using the International Physical Activity Questionnaire Short Form (IPAQ-SF), a self-report questionnaire assessing physical activity over the previous seven-day period [27, 28]. Pre-operative function was assessed using the pre-operative Oswestry Disability Questionnaire (ODQ) score. Pre-operative mobility was categorised into limited or not limited, based on the response to Section 4 (Walking) on the ODQ. A score of three or more indicated limited mobility - unable to walk more than 500 m, requiring a walking aid, or confined to bed.

#### Outcome variables

The outcome variables were described in terms of change between the pre-operative assessment, and six months after surgery. The primary outcome variable was self-reported physical function, measured using the Oswestry Disability Questionnaire (ODQ) [29] and the Short Form 36 Physical Component Summary (SF-36 PCS) (Version 2) [30]. The secondary outcome variables were back and leg pain intensity, measured using the Numeric Pain Rating Scale (NPRS) (0–10) where a higher score indicates more intense pain. The ODQ, SF-36 and the NPRS are commonly used to evaluate outcome following spinal surgery, and have all been validated in back pain populations [31].

At six months participants were also asked to report on their overall satisfaction with their post-operative recovery (Likert scale, 1–5), and if they required any ongoing medical intervention directly related to their surgical procedure.

### Data analysis

Data from the ActivPAL3 were downloaded using PAL Technologies software, and analysed to identify the cumulative amount of time spent walking over the seven-day monitoring period. Descriptive analysis of the data and testing for normality were performed. The four outcome variables (ODQ, SF-36 (PCS), back pain, leg pain) were dichotomised based on the change-score thresholds required to achieve a minimum clinically important difference (MCID) [32, 33] and substantial clinical benefit (SCB) [33] (Table 2). The MCID identifies the change required on a given outcome measure to demonstrate a clinically meaningful improvement in symptoms, and is widely used in lumbar pathology research [32]. The SCB threshold however, identifies the change in outcome required to demonstrate substantial improvement in symptoms [33]. Analysis of outcome based on the MCID and SCB allows for the change in scores to be categorised as no improvement (<MCID), minimal improvement (>MCID, <SCB) and substantial improvement (>SCB). The change in physical function, back pain and leg pain over the six months were analysed using paired *t*-tests, and by using descriptive statistics to examine the proportion of participants who achieved the MCID and SCB thresholds.

**Table 2:** MCID and SCB thresholds

Outcome	MCID (points change)	SCB (points change)
ODQ	10	18.8
SF-36 (PCS)	4.9	6.2
NPRS (back pain)	2	2.5
NRPS (leg pain)	1.6	2.5

*MCID* Minimal Clinically Important Difference, *SCB* Substantial Clinical Benefit, *ODQ* Oswestry Disability Questionnaire, *SF-36* Short Form 36 Physical Component Summary, *NRPS* Numerical Pain Rating Scale

#### Univariate analysis of predictors of outcome

Pearson’s Correlations were performed to calculate the association between the 15 predictor variables and achievement of the SCB threshold for each of the four outcome measures (ODQ; ≥18.8; SF-36 (PCS): ≥6.2; NPRS: ≥2.5) [33].

#### Multivariable logistic regression analysis

The predictor variables that were associated with achieving the SCB threshold at a significance level of *p* < 0.1 in the univariate analysis were entered into a multivariate logistic regression model, using a Backward Wald stepwise method. The threshold of *p <* 0.1 at the univariate stage was chosen to minimise the chance of eliminating potentially predictive variables at this stage [34]. Primary data analysis was conducted using logistic regression analysis and participants with a full data set (no missing outcome or predictor variables).

#### Sensitivity analysis

A secondary sensitivity analysis of the prediction models was performed by a) completing linear regression analysis of the predictor variables that were associated with change in outcome (*p* < 0.1), where change scores were continuous and not dichotomised, and b) using multiple imputation to estimate missing values in both the logistic and linear regression analyses. Comparative analysis of the pre-operative and demographic data of participants excluded due to missing data was performed using *t*-tests.

All analyses were completed in SPSS version 24 (IBM, New York, USA). The target sample size for this study was calculated based on the recommendation of five to ten outcome events per predictor variable (EPV) [35]. It was estimated that SCB would be achieved by 50% of participants on the 15 predictor variables, therefore, the minimum recommended number of participants needed to complete these analyses was 150. It was expected that a total of approximately 300 patients would be admitted for lumbar spinal surgery over the six-month recruitment period. To allow for participant exclusion, variation in EVP, missing data and loss to follow-up, the minimum recruitment target was 250 participants.

## Results

### Participant characteristics

A total of 233 participants consented to participate between April and November 2016. Participant characteristics are presented in Table 3, and a summary of missing data and reasons for exclusion is presented in Table 4. A further 62 participants were lost to follow-up due to incomplete activity monitor data and loss to follow-up, the remaining 171 participants (73.4%) were included in the final analysis. There was no statistically significant difference in demographic or pre-operative data between participants excluded due to incomplete activity monitor data and loss to follow-up, and those included in the final analysis (Additional file 1: Table S1, S2 in Appendix).

**Table 3:** Participant characteristics (N = 233)

	N	(%)
Age		
< 65	119	(51%)
≥ 65	114	(49%)
Sex		
Male	118	(51%)
Female	115	(49%)
Smoking Status		
Non-smoker	214	(92%)
Smoker	18	(8%)
Incomplete data	1	(0.4%)
Obesity		
BMI < 30	154	(66%)
BMI ≥30	73	(31%)
Incomplete data	6	(3%)
Diabetic		
No	207	(89%)
Yes	25	(11%)
Incomplete data	1	(0.4%)
Depression		
No (PHQ-9 < 10)	130	(56%)
Yes (PHQ-9 ≥ 10)	98	(42%)
Incomplete data	5	(2%)
Anxiety		
No (GAD-7 < 10)	159	(68%)
Yes (GAD-7 ≥ 10)	69	(30%)
Incomplete data	5	(2%)
Pre-operative pain duration		
< 12 months	106	(45%)
≥ 12 months	115	(49%)
Incomplete data	12	(5%)
Neurological deficit (self-report)		
No	17	(7%)
Yes	214	(92%)
Incomplete data	2	(1%)
Pre-operative activity (IPAQ-SF)		
Low	130	(56%)
Moderate	64	(27%)
High	29	(12%)
Incomplete data	10	(4%)
Pre-operative mobility (ODQ Section 4)		
Un-restricted (< 3)	113	(48%)
Restricted (≥3)^a^	120	(52%)
Pre-operative function (ODQ category)		
0–20%	15	(6%)
21–40%	93	(40%)
41–60%	85	(36%)
61–80%	37	(16%)
81–100%	2	(1%)
Incomplete data	1	(0.4%)
Surgical procedure		
Decompression	63	(27%)
Discectomy	96	(41%)
Fusion	74	(32%)
Number of vertebral levels		
Single	175	(75%)
Multiple	58	(25%)

BMI Body mass index, PHQ-9 Patient Health Questionnaire 9; GAD-7, Generalised Anxiety Disorder 7-item scale; IPAQ-SF, International Physical Activity Questionnaire Short Form; ODQ, Oswestry Disability Questionnaire; ^a^Restricted mobility: ODQ Section 4, score of ≥3 (unable to walk more than 500 m, or requires a stick, crutches or other support)

**Table 4:** Summary of Included and Excluded Data

	Excluded (N)	Included (N)
Invited to participate:		262
Declined	17	
Excluded		
Unable to provide informed consent	4	
Co-existing condition limiting walking	6	
Withdrew consent	2	
Completed pre-operative OM’s and activity monitoring:		233^a^
Lost/faulty monitor	27	
Incomplete monitoring period	16	
Incomplete outcome measures	19	
Data sets available for multivariable analysis:		171
Included in multivariable analysis^b^:		
ODQ	14	157
SF-36 (PCS)	17	154
Back pain	n/a	n/a
Leg pain	18	196^c^

*OM’s* Outcome measures, *ODQ* Oswestry Disability Questionnaire, *SF-36 (PCS)* Short Form 36 Physical Component Summary

^a^Total number included in sensitivity analysis using multiple imputation to estimate missing data; ^b^Remaining exclusions from individual regression analyses were due to incomplete individual outcome measures or missing independent variable data; ^c^Walking time was not significantly correlated with change in leg pain, the 43 participants with missing activity monitor data included in the multivariable analysis

Preliminary analysis of the accelerometer data revealed that a high proportion of participants had removed the activity monitor during the final 24 h of monitoring. To remove the chance of systematic error as a result of incomplete data, the seventh day of monitoring has been removed from analysis. The total walking time represents the first six post-operative days.

There was a statistically significant improvement in physical function (ODQ and SF-36 PCS), back pain, and leg pain from baseline to six months after surgery (Table 5). Descriptive statistical analysis identified that the proportion of participants who achieved over and above the MCID threshold was between 64% (back pain) and 72% (SF-36 PCS). Between 51% (ODQ) and 66% (SF-36 PCS) of participants exceeded the threshold required to demonstrate a substantial clinical benefit. The average score for overall satisfaction with post-operative recovery was 4.11 (Likert scale, 1–5), with 41% of participants reporting a satisfaction score of 5 (definitely satisfied). Seventeen participants (7%) reported post-operative complications that required ongoing medical intervention (revision/further surgery *N* = 10; infection *N* = 3; deep vein thrombosis N = 1; cerebrospinal fluid leak N = 1; hematoma N = 1; reason not provided N = 1). There was no significant association between the time spent walking and post-operative complications.

**Table 5:** Outcome measurement: preoperative and six months post-operative; achievement of change thresholds

Outcome	Outcome measure	Baseline mean (SD)	Six months mean (SD)	Difference in mean (95%CI)	*t-*statistic, *p-*value	MCID N (%)	SCB N (%)
Physical function	ODQ	44.14 (16.35)	22.37 (17.43)	21.77 (18.44–25.10)	*t =* 15.97*, p* = < 0.01	142 (71%)	102 (51%)
	SF-36 (PCS)	32.89 (7.07)	43.74 (10.03)	−10.85 (−9.12 - -12.60)	*t =* 15.10*, p* = < 0.01	139 (72%)	127 (66%)
Back Pain	NRPS	5.75 (2.78)	2.88 (2.61)	2.87 (2.33–3.40)	*t =* 12.52*, p* = < 0.01	127 (64%)	107 (54%)
Leg Pain	NRPS	5.90 (3.02)	2.25 (2.66)	3.57 (3.08–4.22)	*t =* 14.14*, p* = < 0.01	134 (68%)	114 (58%)

*MCID* Minimal Clinically Important Difference, *SCB* Substantial Clinical Benefit, *ODQ* Oswestry Disability Questionnaire, *SF-36 (PCS)* Short Form 36 Physical Component Summary, *NRPS* Numerical Pain Rating Score

### Univariate analysis

Greater post-operative total walking time was correlated with achieving the SCB threshold on both the ODQ (*p* = 0.05) and the SF-36 (PCS) (*p* = 0.01) (Additional file 1: Table S3 in Appendix). In addition, female sex, pre-operative pain duration of less than 12 months, low pre-operative activity, restricted pre-operative mobility, lower pre-operative function and single-level surgery were correlated with achieving the SCB threshold on the SF-36 (PCS) (*p* < 0.1); An age of less than 65 years, no pre-operative anxiety (indicated by a negative correlation between anxiety and change in outcome, Additional file 1: Table S1 in Appendix) and a pre-operative pain duration of less than 12 months were correlated with achieving the SCB threshold on the SF-36 (PCS) (*p* < 0.1). Female sex, lower pre-operative function and single-level surgery were correlated with achieving the SCB threshold in leg pain scores (*p* < 0.1). Achieving the SCB threshold on the back pain scores was not correlated with any of the predictor variables on univariate analysis.

### Multivariable logistic regression analysis

Greater total walking time over the first six post-operative days was predictive of substantial clinical improvement in physical function at six months (ODQ change of ≥18.8 points). For every one hour increase in total walking time, the odds of achieving the ODQ SCB change threshold increased by 18% (Exp(β) 1.18, 95%CI 1.01–1.37) (Table 6). In addition, a pre-operative pain duration of less than 12 months and lower pre-operative function (higher ODQ score) predicted achievement of the SCB threshold on the ODQ (Table 6).

**Table 6:** Multivariable logistic regression analysis

Outcome measure	Variable	β	Exp (β)	(95% CI)
ODQ	Total post-operative walking time (hours)	0.16	1.18	(1.02–1.37)
	Pre-operative pain duration < 12 months	1.00	2.71	(1.28–5.74)
	Pre-operative function (ODQ categories^a^)	1.39	4.02	(2.33–6.93)
SF-36 (PCS)	Age < 65	0.86	2.36	(1.14–4.85)
	Pre-operative pain duration < 12 months	1.26	3.52	(1.69–7.33)

Interpretation of results: Exp (β) is equivalent to the odds ratio (OR). The predictor variables may be applied to determine the odds of achieving the SCB threshold for the given outcome measure. For example, for each additional hour of walking the odds of achieving ODQ SCB increases by 18%; for a participant less than 65 years old, the odds of achieving the SF-36 (PCS) SCB is 2.36 greater than those 65 years or over

ODQ, Oswestry Disability Questionnaire; SF-36 (PCS), Short Form 36 Physical Component Summary; ^a^ODQ categories: 1: 0–20, 2: 21–40, 3: 41–59, 4: 60–79, 5: 80–100 (reference value: ODQ score 0–20)

Total walking time was not predictive of achieving the SCB change threshold on the SF-36 (PCS) or leg pain scores at six months. An age of less than 65 years old, and pre-operative pain of less than 12 months predicted achievement of the SCB threshold (Table 6). There were no significant predictors for achieving the SCB change threshold for back or leg pain.

### Sensitivity analysis

Total walking time remained a significant predictor of functional recovery (change in ODQ score from pre-operative to six months post-operative) using a linear regression model (B = 1.54; 95%CI 0.60–2.48) (Additional file 1: Table S4 in Appendix). When using multiple imputation models to estimate missing data, total walking time was not a significant predictor of functional recovery or change in pain scores, using either logistic or linear regression analysis (Additional file 1: Table S5, S6 in Appendix).

## Discussion

This study found that greater walking time in the first post-operative week was associated with substantial improvement in self-reported function (ODQ), six months after lumbar surgery. In addition to walking time, experiencing pain for 12 months or less prior to surgery, poor pre-operative physical function and being younger than 65 years were associated with substantial improvement in function on the ODQ and/or the SF-36 (PCS) at six months.

The association between greater post-operative walking time and improved physical function has several implications. Greater walking time early after surgery may be an early indication of a successful surgical intervention, resulting in greater longer-term functional recovery. These patients with a greater walking time may therefore need less formal post-operative rehabilitation. Conversely, patients with a lower post-operative walking time may require more intensive rehabilitation to achieve substantial improvement in physical function. However, while this research suggests that the current focus of physiotherapy intervention on walking may be justified [10–12], further research is required to determine whether providing intervention targeted towards increasing walking time early after surgery then leads to improved longer-term outcome.

There was little overlap in the variables that predicted substantial improvement between the two measures of physical function, which likely reflects the differences in construct between the two measurement tools. Greater walking time was predictive of functional recovery on the ODQ but not the SF-36 (PCS). As the ODQ is designed to directly measure the impact back pain related disability has on physical function [36] it is unsurprising that walking, a form of physical activity, was associated with greater change on the ODQ. In contrast, the physical component summary of the SF-36 is designed to assess physical function in relation to Health Related QOL (HRQOL), and takes into account physical role, bodily pain, general health and mental health, in addition to physical function [37]. This broader definition may explain why alterations to physical activity in isolation did not influence change on the SF-36 (PCS). These results suggest that the ODQ and the SF-36 (PCS) measure distinctly different constructs in this population, a finding consistent with previous research [38].

No patient variables were found to be predictive of substantial improvement in back or leg pain in the multivariable logistic regression model, however on sensitivity analysis restricted pre-operative mobility was predictive of improvement in back-pain scores, and female sex was predictive of improvement in leg pain scores. Sensitivity analysis was conducted using linear regression analysis, where the change in pain from pre to post-operative was assessed as a continuous score rather than dichotomized based on the substantial clinical benefit threshold. This discrepancy may indicate that while restricted pre-operative mobility and female sex are significantly associated with improvement in pain scores, the change in pain over time may not represent a clinically meaningful improvement.

The overall change in outcome after lumbar surgery reported in this study are comparable with previously published results [7, 39]. While the majority of patients achieved the threshold required to demonstrate substantial improvement in function and pain, around a third did not achieve a minimal clinically important change in outcome. As the substantial clinical benefit threshold was used to dichotomize the data for the logistic regression analysis, we can be confident that these results represent clinically meaningful change, which is of particular importance as the benefit of surgical intervention for lumbar spine pathology remains inconclusive [4–6]. These findings do however emphasize the need for further investigation into interventions designed to optimize longer term recovery of physical function.

### Limitations

There are several limitations to this study that need to be considered when interpreting these results. Up to 34% of participants who completed the monitoring period were not included in the multivariable analysis, and exclusion of these participants may limit how well the final sample reflects the broader population. However, as there was no statistically significant difference in the pre-operative or demographic variables between the participants that were included in the final analysis and those that were not, we are confident that these findings are representative of a larger sample.

As this research was conducted at a single site, the external validity of this study may be limited. However, patients of 11 different surgeons were included in this study all with differing post-operative protocols, and the presenting characteristics of the patients were consistent with those reported by other spinal surgery studies [7]. Further research across additional settings and patient populations would increase the confidence that these findings are more broadly generalizable.

The aim of this study was to investigate the direct association between patient variables and recovery of physical function after lumbar surgery with routine peri-operative care. Patient care was not standardised or modified in any way from usual care in the hospital setting, and the study was not designed to investigate a specific therapy or exercise intervention. Regression analysis was therefore most suited to examine the direct associations between patient variables and recovery after surgery. It is possible that parameters of the individual peri-operative rehabilitation program, such as the type, intensity, frequency or duration of exercise may have directly influenced outcome at six months, or interacted with the patient variables being examined in this study to indirectly impact outcome. However, our study was not designed to explore more complex predictive models such as an omnibus test of mediation, and as such the parameters of peri-operative rehabilitation were not recorded. Now that a direct association between walking and post-operative outcomes has been established in our study, future research needs to examine the mechanisms driving this relationship by means of more complex models such as mediation analysis.

## Conclusion

This study found that people who spend more time walking in the week after lumbar surgery were more likely to experience substantial improvement in physical function at six months, as measured on the ODQ. Further research is required to ascertain whether intervention designed to increase walking early after lumbar surgery leads to improved recovery of function.

## Additional file

Additional file 1: **Table S1.** Characteristics of Included and Excluded Participants (continuous data, independent *t-*tests). **Table S2.** Characteristics of Included and Excluded Participants (dichotomous/categorical data, Chi Squared). **Table S3.** Univariate analysis – correlation between predictor variables and achieving the SCB change threshold. **Table S4.** Sensitivity analysis. Linear regression analysis – change in outcome from pre-operative to six months post-operative. **Table S5.** Sensitivity analysis. Logistic regression analysis (multiple imputation) – achievement of SCB threshold. **Table S6.** Sensitivity analysis. Linear regression analysis (multiple imputation) – change in outcome from pre-operative to six months post-operative. (DOCX 40 kb)

## Data Availability

The datasets used and/or analysed during the current study are available from the corresponding author on reasonable request.

## Abbreviations

BMI: Body Mass Index
EVP: Events per Predictor Variable
GAD-7: Generalized Anxiety Disorder 7-item Scale
HRQOL: Health Related Quality of Life
IPAQ-SF: International Physical Activity Questionnaire Short Form
MCID: Minimum Clinically Important Difference
NPRS: Numerical Pain Rating Scale
ODQ: Oswestry Disability Questionnaire
OM: Outcome Measure
OR: Odds Ratio
PHQ-9: Patient Health Questionnaire Depression Scale
SCB: Substantial Clinical Benefit
SF-36 (PCS): Short Form 36 Physical Component Summary
SVPHM: St Vincent’s Private Hospital Melbourne

## References

[1] Bae HW, Rajaee SS, Kanim LE (2013). Nationwide trends in the surgical management of lumbar spinal stenosis. Spine.

[2] Harris IA, Dao AT (2009). Trends of spinal fusion surgery in Australia: 1997 to 2006. ANZ J Surg.

[3] Kobayashi K, Ando K, Nishida Y (2018). Epidemiological trends in spine surgery over 10 years in a multicenter database. Eur Spine J.

[4] Jacobs WC, van Tulder M, Arts M (2011). Surgery versus conservative management of sciatica due to a lumbar herniated disc: a systematic review. Eur Spine J.

[5] Phillips FM, Slosar PJ, Youssef JA (2013). Lumbar spine fusion for chronic low back pain due to degenerative disc disease: a systematic review. Spine.

[6] Zaina F, Tomkins-Lane C, Carragee E, Negrini S (2016). Surgical versus non-surgical treatment for lumbar spinal stenosis. Cochrane Database of Syst Rev.

[7] Weinstein JN, Tosteson TD, Lurie JD, Tosteson A, Blood E, Herkowitz H, Cammisa F, Albert T, Boden SD, Hilibrand A, Goldberg H, Berven S, An H (2010). Surgical versus non-operative treatment for lumbar spinal stenosis four year results of the spine patient outcomes research trial (SPORT). Spine.

[8] Gilmore SJ, McClelland JA, Davidson M (2015). Physiotherapeutic interventions before and after surgery for degenerative lumbar conditions: a systematic review. Physiother..

[9] Oosterhuis T, Costa LOP, Maher CG, de Vet HCW, van Tulder MW, Ostelo RWJG. Rehabilitation after lumbar disc surgery. Cochrane Database Syst Rev. 2014;3. 10.1002/14651858.CD003007.pub3. 10.1002/14651858.CD003007.pub3 PMC7138272 24627325

[10] Gilmore SJ, McClelland JA, Davidson M (2016). Physiotherapy management of patients undergoing lumbar spinal surgery for degenerative conditions: a survey of Australian physiotherapists. N Z J Physiother.

[11] Rushton A, Heneghan N, Heap A (2014). Survey of current physiotherapy practice for patients undergoing lumbar spinal fusion in the UK. Spine.

[12] Williamson E, White L, Rushton A (2007). A survey of post-operative management for patients following first time lumbar discectomy. Eur Spine J.

[13] Mancuso CA, Duculan R, Graig CM, Girardi FP (2018). Psychosocial variables contribute to length of stay and discharge destination after lumbar surgery independent of demographic and clinical variables. Spine J.

[14] Hey HWD, Luo N, Chin SY, Lau ETC, Wang P, Kumar N, Lau L, Ruiz JN, Thanbiah JS, Lui KG, Wong H (2018). The predictivive value of preoperative health-related quality-of-life scores on postoperative patient-reported outcome scores in lumbar spine surgery. Global Spine J.

[15] Kanaan SF, Arnold PM, Burton DC, Yeh H, Loyd L, Sharma NK (2015). Investigating and predicting early lumbar spine surgery outcomes. J Allied Health.

[16] Pearson A, Lurie J, Tosteson T, Zhao W, Abdu W, Weinstein J (2012). Who should have surgery for spinal stenosis?: treatment effect predictors in SPORT. Spine..

[17] Appaduray SP, Lo P (2013). Effects of diabetes and smoking on lumbar spinal surgery outcomes. J Clin Neurosci.

[18] Archer KR, Seebach CL, Mathis SL, Riley Iii LH, Wegener ST (2014). Early postoperative fear of movement predicts pain, disability, and physical health six months after spinal surgery for degenerative conditions. Spine J.

[19] Gilmore SJ, Davidson M, Hahne AJ, McClelland JA. The validity of using activity monitors to detect step count after lumbar fusion surgery. Disabil Rehabil. 2018. 10.1080/09638288.2018.1509140. 10.1080/09638288.2018.1509140 30326754

[20] Gilmore SJ, Hahne AJ, Davidson M, McClelland JA (2019). Physical activity pattersons after lumbar spinal surgery. Disabil Rehabil.

[21] Mancuso CA, Duculan R, Girardi FP (2017). Healthy physical activity levels below recommended thresholds two years after lumbar spine surgery. Spine..

[22] Smuch M, Muaremi A, Zheng P, Norden J, Sinha A, Hu R, Tomkins-Lane C (2018). Objective measurement of function following lumbar spinal stenosis decompression reveals improved functional capacity with stagnant real-life physical activity. Spine J.

[23] Gilmore S, McClelland JA, Davidson M (2016). Does walking after lumbar spinal surgery predict recovery of function at six months? Protocol for a prospective cohort study. BMC Musculoskelet Disord.

[24] Kroenke K, Spitzer RL, Williams JBW (2001). The PHQ-9. J Gen Intern Med.

[25] Choi Y, Mayer TG, Williams MJ, Gatchel RJ (2014). What is the best screening test for depression in chronic spinal pain patients?. Spine J.

[26] Spitzer RL, Kroenke K, Williams JB, Lowe B (2006). A brief measure for assessing generalized anxiety disorder: the GAD-7. Arch Intern Med.

[27] Craig CL, Marshall AL, Sjostrom M, Bauman AE, Booth ML, Ainsworth BE, Pratt M, Ekelund U, Yngve A, Sallis JF, Oja P (2003). International physical activity questionnaire: 12-country reliability and validity. Med Sci Sports Exerc.

[28] Helmerhorst HJ, Brage S, Warren J, Besson H, Ekelund U (2012). A systematic review of reliability and objective criterion-related validity of physical activity questionnaires. Int J Behav Nutr Phys Act.

[29] Davidson M, Keating JL (2002). A comparison of five low back disability questionnaires: reliability and responsiveness. Phys Ther.

[30] Ware JEJP, Sherbourne CDP (1992). The MOS 36-ltem short-form health survey (SF-36): I. conceptual framework and item selection. Med Care.

[31] Chapman JR, Norvell DC, Hermsmeyer JT (2011). Evaluating common outcomes for measuring treatment success for chronic low Back pain. Spine..

[32] Ostelo RW, Deyo RAP, Stratford PP (2008). Interpreting change scores for pain and functional status in low Back pain: towards international consensus regarding minimal important change. Spine..

[33] Glassman SD, Copay AG, Berven SH (2008). Defining Substantial Clinical Benefit Following Lumbar Spine Arthrodesis. J Bone Joint Surg Am.

[34] Park J, Kim D, Seo D (2019). Predictors of response to a medical branch block: MRI analysis of the lumbar spine. J Clin Med.

[35] Vittinghoff E, McCulloch CE (2007). Relaxing the rule of ten events per variable in logistic and cox regression. Am J Epidemiol.

[36] Fairbank J, Couper J, Davies JB, O’Brien JP (1980). The Oswestry low Back pain questionnaire. Physiother..

[37] Ware J, Kosinski M (2008). SF-36v2 health survey: administration guide for clinical trial investigators.

[38] Bombardier C (2000). Outcome assessments in the evaluation of treatment of spinal disorders: summary and general recommendations. Spine..

[39] Fritsch CG, Ferreira ML, Maher CG (2017). The clinical course of pain and disability following surgery for spinal stenosis: a systematic review and meta-analysis of cohort studies. Eur Spine J.

