# Supplementary material for: Predictors of substantial improvement in physical function six months after lumbar surgery: is early post-operative walking important? A prospective cohort study
Source: BMC Musculoskelet Disord. 2019 Sep 11;20:418. doi: 10.1186/s12891-019-2806-7 (PMC6737667; doi:10.1186/s12891-019-2806-7)
Supplement: Supplementary file 1 — Table S1. Characteristics of Included and Excluded Participants (continuous data, independent t-tests). Table S2. Characteristics of Included and Excluded Participants (dichotomous/categorical data, Chi Squared). Table S3. Univariate analysis – correlation between predictor variables and achieving the SCB change threshold. Table S4. Sensitivity analysis. Linear regression analysis – change in outcome from pre-operative to six months post-operative. Table S5. Sensitivity analysis. Logistic regression analysis (multiple imputation) – achievement of SCB threshold. Table S6. Sensitivity analysis. Linear regression analysis (multiple imputation) – change in outcome from pre-operative to six months post-operative. (DOCX 40 kb) [file 12891_2019_2806_MOESM1_ESM.docx]

Table S1: Characteristics of Included and Excluded Participants (continuous data, independent *t-*tests)

|  | Included  Mean (SD) | Excluded  Mean (SD) | Difference in mean (95%CI) | *t*-statistic, *p-*value |
| --- | --- | --- | --- | --- |
| Age (years) | 61.59 | 60.64 | 0.96 (-3.25 – 5.17) | *t*=0.45, *p=*0.65 |
| BMI | 28.43 | 28.10 | 0.33 (-1.32 – 1.98) | *t*=0.40, *p=*0.69 |
| Pre-operative pain duration (months) | 40.80 | 30.82 | 9.98 (-12.94 – 32.90) | *t*=0.86, *p=*0.39 |
| Pre-operative function (ODQ) | 44.50 | 43.83 | 0.67 (-4.10 – 5.42) | *t*=-0.27, *p=*0.78 |
| Pre-operative SF36 (PCS) | 32.77 | 34.10 | 1.33 (-0.82 – 3.50) | *t*=-1.22, *p=*0.22 |
| Pre-operative SF36 (MCS) | 46.94 | 45.61 | 1.34 (-2.31 – 4.97) | *t*=0.72, *p=*0.47 |
| Pre-operative back pain (NPRS, 0-10) | 5.76 | 6.10 | 0.34 (-0.46 – 1.14) | *t*=-0.84, *p=*0.40 |
| Pre-operative leg pain (NPRS (0-10) | 5.77 | 6.27 | 0.50 (-0.40 – 1.40) | *t*=-1.10, *p=*0.27 |

BMI Body mass index, ODQ, Oswestry Disability Questionnaire;), SF36 Short Form 36, PCS Physical component Summary, MCS Mental Component Summary, NPRS Numerical Pain Rating Scale

Table S2 Characteristics of Included and Excluded Participants (dichotomous/categorical data, Chi Squared)

|  | Included  N (%) | | Excluded  N (%) | | Chi squared | | *p-*value | |
| --- | --- | --- | --- | --- | --- | --- | --- | --- |
| Sex |  | |  | |  | |  | |
| Male | 83 (48%) | | 35 (57%) | | 1.50 | | *p=*0.22 | |
| Female | 89 (52%) | | 26 (43%) | |  | |  | |
| Smoking Status |  | |  | |  | |  | |
| Non-smoker | 157 (91%) | | 57 (95%) | | 0.86 | | *p=*0.35 | |
| Smoker | 15 (9%) | | 2 (5%) | |  | |  | |
| Diabetic |  | |  | |  | |  | |
| No | 155 (90%) | | 52 (87%) | | 0.55 | | *p=*0.46 | |
| Yes | 17 (10%) | | 8 (13%) | |  | |  | |
| Depression |  | |  | |  | |  | |
| No (PHQ-9 <10) | 96 (57%) | | 34 (57%) | | 0.004 | | *p=*0.95 | |
| Yes (PHQ-9 ≥10) | 72 (43%) | | 26 (43%) | |  | |  | |
| Anxiety |  | |  | |  | |  | |
| No (GAD-7 <10) | 117 (70%) | | 42 (70%) | | 0.003 | | *p=*0.96 | |
| Yes (GAD-7 ≥10) | 51 (30%) | | 18 (30%) | |  | |  | |
| Neurological deficit (self-report) |  | |  | |  | |  | |
| No | 12 (7%) | | 5 (8%) | | 0.113 | | *p=*0.74 | |
| Yes | 159 (93%) | | 55 (92%) | |  | |  | |
| Pre-operative activity (IPAQ-SF) |  | |  | |  | |  | |
| Low | 102 (61%) | | 28 (49%) | | 2.94 | | *p=*0.23 | |
| Moderate | 43 (26%) | | 21 (37%) | |  | |  | |
| High | 21 (13%) | | 8 (8%) | |  | |  | |
| Pre-operative mobility (ODQ Section 4) | |  | |  | |  | |  |
| Un-restricted (<3) | 84 (49%) | | 29 (48%) | | 0.03 | | *p=*0.86 | |
| Restricted (≥3)^a^ | 88 (51%) | | 32 (52%) | |  | |  | |
| Surgical procedure |  | |  | |  | |  | |
| Decompression | 44 (26%) | | 19 (32%) | | 2.98 | | *p=*0.23 | |
| Discectomy | 68 (40%) | | 28 (46%) | |  | |  | |
| Fusion | 60 (35%) | | 14 (23%) | |  | |  | |
| Number of vertebral levels |  | |  | |  | |  | |
| Single | 131 (76%) | | 44 (72%) | | 0.39 | | *p=*0.53 | |
| Multiple | 41 (24%) | | 17 (28%) | |  | |  | |

PHQ-9 Patient Health Questionnaire 9; GAD-7, Generalised Anxiety Disorder 7-item scale; IPAQ-SF, International Physical Activity Questionnaire Short Form; ODQ, Oswestry Disability Questionnaire; ^a^Restricted mobility: ODQ Section 4, score of ≥ 3 (unable to walk more than 500m, or requires a stick, crutches or other support)

Table S3: Univariate analysis – correlation between predictor variables and achieving the SCB change threshold

|  | Function (ODQ) | | Function^a^ (SF-36 PCS) | | Back Pain (NPRS) | | Leg Pain (NPRS) | |
| --- | --- | --- | --- | --- | --- | --- | --- | --- |
|  | *r* | *p* | *r* | *p* | *r* | *p* | *r* | *p* |
| Total walking time (hours) | 0.152^*^ | 0.048 | 0.196^*^ | 0.012 | -0.062 | 0.421 | 0.059 | 0.450 |
| Age <65years | 0.065 | 0.358 | 0.195^**^ | 0.006 | 0.006 | 0.931 | 0.027 | 0.712 |
| Sex (female) | 0.156^*^ | 0.028 | -0.016 | 0.830 | 0.107 | 0.133 | 0.145^*^ | 0.043 |
| Smoker | -0.007 | 0.917 | -0.048 | 0.509 | 0.052 | 0.468 | 0.051 | 0.475 |
| Pre-operative obesity (BMI<30) | 0.081 | 0.267 | 0.043 | 0.560 | 0.079 | 0.276 | 0.002 | 0.977 |
| Pre-operative diabetes | 0.055 | 0.438 | -0.115 | 0.111 | -0.010 | 0.893 | 0.028 | 0.694 |
| Depression | 0.057 | 0.425 | -0.098 | 0.181 | 0.104 | 0.147 | 0.090 | 0.213 |
| Anxiety | 0.074 | 0.304 | -0.138 | 0.058 | 0.057 | 0.430 | -0.019 | 0.798 |
| Pre-operative pain duration <12 months | 0.238^**^ | 0.001 | 0.255^**^ | 0.000 | -0.018 | 0.805 | 0.081 | 0.268 |
| Pre-operative neurological deficit | 0.044 | 0.542 | 0.025 | 0.729 | 0.078 | 0.277 | 0.043 | 0.550 |
| Low pre-operative activity | 0.190^**^ | 0.008 | -0.034 | 0.647 | 0.019 | 0.797 | 0.055 | 0.454 |
| Restricted pre-operative mobility^b^ | 0.296^**^ | 0.000 | -0.029 | 0.690 | 0.093 | 0.191 | 0.058 | 0.418 |
| Lower pre-operative function | 0.447^**^ | 0.000 | -0.040 | 0.581 | 0.075 | 0.293 | 0.140 | 0.050 |
| Surgical procedure | -0.028 | 0.692 | -0.059 | 0.412 | -0.097 | 0.172 | -0.093 | 0.193 |
| Single-level surgery | 0.119 | 0.093 | 0.026 | 0.716 | 0.055 | 0.441 | 0.127 | 0.075 |

**p*<0.05; ***p*<0.01.

Interpretation of results: Positive *r* values represent a greater chance of achieving the SCB threshold for each outcome when participants present with the listed characteristic, while negative *r* values represent a reduced chance of achieving the SCB threshold.

SCB, Substantial Clinical Benefit; ODQ, Oswestry Disability Questionnaire; SF-36 (PCS), Short Form 36 Physical Component Summary; NRPS, Numerical Pain Rating Scale; BMI, Body mass index

^a^Quality of Life related to physical function; ^b^Restricted pre-operative mobility: ODQ Section 4, score of ≥ 3 (unable to walk more than 500m, or requires a stick, crutches or other support)

Table S4: Sensitivity analysis

Table S4 Sensitivity analysis. Linear regression analysis – change in outcome from pre-operative to six months post-operative

| Outcome | Variable | β | (95%CI) | *p* |
| --- | --- | --- | --- | --- |
| Function (ODQ) | Total walking time (hrs) | 1.54 | (0.60-2.48) | <0.01 |
|  | Pre-operative pain duration (months) | -0.04 | (-0.07- -0.01) | <0.01 |
|  | Pre-operative function (ODQ, 0-100) | 0.61 | (0.46-0.77) | <0.01 |
| Function (SF-36 PCS) | Age (years)^a^ | 0.16 | (0.05-0.28) | <0.01 |
| Back Pain (NPRS) | Restricted pre-operative mobility^b^ | 0.96 | (0.06-1.86) | 0.04 |
| Leg Pain (NRPS) | Sex (Female) | 1.13 | (0.15-2.11) | 0.03 |

Interpretation of results: For each unit increase of a predictor variable, β represents the concurrent change in OM score. For example, each additional hour walked would result in 1.54 points more improvement in the ODQ; participants with restricted pre-operative mobility would have a change in back pain of 0.96 points more on the NPRS than those with unrestricted mobility.

ODQ, Oswestry Disability Questionnaire; SF-36 PCS, Short Form 36 Physical Component Summary; NRPS, Numerical Pain Rating Scale;

^a^Negative change on the SF-36 (PCS) indicates improved function over time - improved function is associated with decreasing age; ^b^Restricted mobility: ODQ Section 4, score of ≥ 3 (unable to walk more than 500m, or requires a stick, crutches or other support)

Table S5 Sensitivity analysis. Logistic regression analysis (multiple imputation) – achievement of SCB threshold

| Outcome measure | Variable | β | Exp (β) | (95%CI) |
| --- | --- | --- | --- | --- |
| ODQ | Pre-operative Pain Duration <12months | 1.00 | 2.71 | (1.45-5.06) |
|  | Pre-operative function (ODQ categories^a^) | 1.33 | 3.77 | (2.52-5.87) |
| SF-36 (PCS) | Age <65 | 0.66 | 1.94 | (1.02-3.68) |
|  | Pre-operative Pain Duration <12months | 0.90 | 2.46 | (1.32-4.58) |

Note: No significant predictive variables for change in back or leg pain.

Interpretation of results: Exp (β) is equivalent to the odds ratio (OR). These variables may be applied to determine the odds of achieving the SCB threshold for the given outcome measure. For example, for a patient less than 65 years old, the odds of achieving the SF-36 (PCS) SCB is 1.94 greater than a patient 65 years or over.

SCB, Substantial Clinical Benefit; ODQ, Oswestry Disability Questionnaire; SF-36 (PCS), Short Form 36 Physical Component Summary;

^a^ODQ categories: 1: 0-20, 2: 21-40, 3: 41-59, 4: 60-79, 5: 80-100.

Table S6 Sensitivity analysis. Linear regression analysis (multiple imputation) – change in outcome from pre-operative to six months post-operative

| Outcome measure | Variable | β | (95%CI) | *p* |
| --- | --- | --- | --- | --- |
| ODQ | Pre-operative pain duration (months) | -0.03 | (-0.70- -0.10) | 0.01 |
|  | Pre-operative function (ODQ, 0-100) | 0.63 | (0.49-0.76) | <0.01 |
|  | Single level surgery | -5.75 | (-10.63—0.868) | 0.02 |
| SF-36 (PCS) | Age (years)^a^ | 0.14 | (0.02-0.27) | 0.03 |

Interpretation of results: For each unit increase of the predictor variable, β represents the concurrent change in OM score. For example, for each additional month of pre-operative pain the change in ODQ would decrease (negative association) by 0.03 points.

Note: No significant predictive variables for change in back or leg pain.

ODQ, Oswestry Disability Questionnaire; SF-36 (PCS), Short Form 36 Physical Component Summary;

^a^Negative change on the SF-36 (PCS) indicates improved function over time - improved function is associated with decreasing age.
